# Effects of neutral detergent fiber levels on apparent nutrient digestibility and intestinal microbiota composition and function in forest musk deer

**DOI:** 10.3389/fvets.2025.1658189

**Published:** 2025-09-11

**Authors:** Qindan Dai, Yu He, Jie Wu, Lei Zhou, Guimei Jiang, Feng Chen

**Affiliations:** ^1^Sichuan Institute for Drug Control (Sichuan Testing Center of Medical Devices, Sichuan Institute of Musk Deer Breeding), Chengdu, China; ^2^Chengdu Century Investment Co., Ltd., Chengdu, China

**Keywords:** forest musk deer, neutral detergent fiber, nutrient digestibility, metagenome, gut microflora

## Abstract

This experiment was conducted to explore the effects of different neutral detergent fiber (NDF) levels on nutrient apparent digestibility and intestinal microbiota composition and function in adult male forest musk deer (FMD) (*Moschus berezovskii*). A total of 18 adult male forest musk deer (FMD) (aged 4–10 years) with an initial average body weight of 7.09 ± 0.82 kg were selected and randomly divided into three groups with different NDF levels: L: 21.60%, M: 25.14%, and H: 28.47%. The FMD were used in a 50-day trial. The results showed that the apparent digestibility of NDF and acid detergent fiber (ADF) first increased and then decreased as NDF levels rose, with the M group showing the highest digestibility (*p* < 0.05). The H group exhibited significantly higher (*p* < 0.05) Chao1 and ACE indices compared to the L group. In addition, at the phylum level (the relative abundance > 0.5%), no significant differences were observed among the three groups, except for Mycoplasmatota, which showed higher (*p* < 0.05) relative abundance in the M group compared to the L group. At the genus level (the relative abundance > 1%), the three groups did not change (*p* > 0.05) significantly. In the KEGG function analysis, differentially expressed genes were primarily enriched in pathways related to organismal systems and human diseases. In the CAZy functional analysis, significant differences (*p* < 0.05) were observed in glycoside hydrolases (GHs) and carbohydrate-binding modules (CBMs), with the M group showing clear enrichment in fiber-degrading enzymes. Overall, the M group demonstrated superior NDF apparent digestibility and enhanced fiber degradation capacity. Therefore, a dietary NDF level of approximately 25% is recommended as optimal for adult male FMD.

## 1 Introduction

The forest musk deer (FMD) (*Moschus berezovskii*), the smallest species within the *Moschus* genus, is a ruminant under first-class protection in China (1). The musk secreted by male forest musk deer (FMD) holds great economic and social value, particularly in the production of high-end perfumes and traditional Asian medicine (2, 3). However, due to long-term illegal hunting and habitat fragmentation over the past 70 years—especially during the 1980s—the number of FMD has sharply declined (4). Although China initiated an artificial musk deer farming program in 1958, more than 60 years later (5), the captive breeding of FMD continues to face substantial challenges due to the species’ physiological constraints. For example, their heightened susceptibility to stress potentially contributes to elevated captive morbidity (35 ~ 62%) and mortality rates (18 ~ 30%), primarily due to gastrointestinal and respiratory diseases (6). Additionally, there are critical knowledge gaps regarding the nutritional requirements and feeding standards of FMD. This complex issue has resulted in markedly slower progress in breeding technology compared to other ungulate species (cattle, sheep, and deer, among others). Current husbandry practices for FMD remain largely experience-based, lacking scientific validation regarding optimal dietary composition. The nutritional adequacy of diets for captive FMD remains uncertain, potentially posing the risks of both deficiency and excess. Generally, wild FMD exhibit seasonal dietary diversity through selective browsing of varied plant species, but captive FMD are typically fed a restricted diet consisting primarily of concentrates formulated from conventional ruminant feedstuffs, supplemented with a limited variety of leaves and vegetables obtained through manual collection. However, this feeding approach lacks established nutritional standards, making it difficult to determine whether it meets the nutritional requirements or increases the body’s burden for FMD. Meanwhile, the nutritional requirements of FMD cannot be fully based on those of other ruminants because of special feeding ecology. This nutritional paradigm gap may contribute to the high incidence of digestive disorders in captive FMD, and appropriate nutritional provision is critical for the physiological maintenance and growth of captive FMD.

Optimal nutritional levels are essential for establishing stable gut microbial communities, which, in turn, promote host health. Gong et al. (7), through a comprehensive analysis of gut microbiome composition, determined that growing male FMD require a dietary protein level of 13.37%. Even so, reports on the nutritional requirements of FMD remain scarce. Neutral detergent fiber (NDF) serves as a critical nutritional indicator in ruminant feeding systems; suitable NDF levels are essential for ruminal health maintenance, production performance, high feed intake, and digestibility (8, 9). Given the stringent conservation policies governing FMD husbandry in China—which strictly prohibit any invasive procedures that may cause harm or stress—this study adopted non-invasive fecal sampling to evaluate the effects of varying NDF levels, using nutrient digestibility and fecal microbiome profiling indices. Nutrient digestibility coefficients serve as fundamental indicators of feeding efficiency in FMD, directly reflecting the animal’s capacity to utilize dietary components. The gut microbiome serves as a critical interface between diet and host physiology, with particular relevance for FMD conservation, and is frequently studied in forest musk deer research. Furthermore, fecal microbial profiles provide multidimensional insights into health status, immune competence, nutritional adaptation, and evolutionary fitness (10, 11). Therefore, this study systematically evaluated the effects of dietary NDF levels on nutrient apparent digestibility and the structure and function of intestinal microbiota in FMD. The findings recommend the appropriate NDF levels for the diet of adult male FMD to provide a valuable reference for scientifically formulating their nutritional plan.

## 2 Materials and methods

### 2.1 Ethics committee approval

All experimental procedures involving animals were reviewed and approved by the Institutional Animal Care and Use Committee at the Sichuan Institute of Musk Deer Breeding, Chengdu, Sichuan, China (SCYS-E2024002). All studies were conducted in accordance with the guidelines outlined in the Ethical Treatment of Experimental Animals of China.

### 2.2 Experimental design and animals

This study was conducted at the Musk Deer Farm of the Sichuan Institute of Musk Deer Breeding, located in Marcon County, Aba Tibetan and Qiang Autonomous Prefecture, Sichuan Province, China. A total of 18 adult male FMD (years 4–10) with similar body conditions (weighing 7.09 ± 0.82 kg) were selected. These FMD were randomly divided into three groups, with six replicates per group and one FMD in each replicate. The experiment lasted for 50 days. All animals were housed individually, and their sheds were thoroughly cleaned before the experiment.

### 2.3 Diet and feeding management

In this experiment, each FMD was fed the total mixed ration (TMR) at 9:00 a.m. and 16:00 p.m. every day. Considering the physiological activity patterns of FMD (crepuscular habits), only 20–30% of the total daily feed ration was provided in the morning, with the remainder fed in the afternoon. The TMR consists of dried leaves (a mixture of several kinds of leaves), concentrate, and fresh vegetables, and the leaves and vegetables were pre-treated by cutting them into pieces approximately 1–3 cm in size before mixing. The NDF contents of the three TMR were 21.6% (L group), 25.14% (M group), and 28.47% (H group), respectively. The dietary composition of the experimental groups is presented in Table 1. All FMD were provided with ration and water ad libitum, and the cleanliness of the enclosure was maintained.

**Table 1:** Composition and nutritional levels of the experimental diet (DM basis).

Item	Group		
	L	M	H
Ingredients (%)			
Dry leaves	30	40	35
Alfalfa meal	1	6	20
Succulent feed^a^	19	14	15
Concentrate^b^	50	40	30
Total	100	100	100
Nutrient levels^c^			
CP (%)	17.09	16.59	16.23
NDF (%)	21.60	25.14	28.47
ADF (%)	13.43	17.61	20.42
EE (%)	6.24	6.06	5.82
GE (MJ/kg)	16.46	16.53	16.32
Ash (%)	9.05	8.98	9.34
Calcium (%)	1.06	1.21	1.33
Phosphorus (%)	0.49	0.43	0.40

L = low NDF (21.6%); M = middle NDF (25.14%); H = high NDF (28.47%); CP = crude protein; NDF = neutral detergent fiber; ADF = acid detergent fiber; EE = ether extract; GE = gross energy.

a The succulent feed composition (on a fresh weight basis) consisted of 55% lettuce, 15% carrot, 15% pumpkin, and 15% cabbage.

b The concentrate composition (on an air-dry basis) consisted of 57% corn, 19.5% soybean meal, 10% bran, 7% extruded soybean, 2.5% Angel yeast, 1.5% sodium bicarbonate, and 2.5% premix.

c Nutrient levels were based on measured values.

### 2.4 Data and sample collection

On day 16 of the experimental period, daily feed provision and residues were recorded for each FMD, with refusal samples collected daily. Then, the samples were dried at 105 °C for 4 h in a forced-air oven, equilibrated in a desiccator, and weighed for DMI analysis.

A total of 0.5 kg of the TMR from the different experimental groups was collected, packed into ziplock bags, marked, and stored in the refrigerator at −20 °C for later use. After mixing the dietary samples collected from each group, samples of appropriate portions were obtained and dried to a constant weight in an oven at 105 °C. The dried samples were then ground using a pulverizer and passed through a 40-mesh sieve for further analysis.

During days 43–49, fresh feces from all FMD were collected into ziplock bags by the total fecal collection method for the digestibility test. Fecal samples were collected and pooled based on a per-FMD individual basis. A representative 10% aliquot was obtained from the thoroughly mixed fecal matter and homogenized with a 10% sulfuric acid solution at a ratio of 10 mL acid per 100 g of the fecal sample. These samples were then stored with the residual fecal samples at −20 °C for subsequent determination of apparent nutrient digestibility.

On the final experimental day, fresh fecal samples were collected prior to morning feeding, which were immediately snap-frozen in liquid nitrogen and stored at −80 °C for subsequent metagenomic analysis.

### 2.5 Nutrient digestibility and digestive energy

The feed and fecal samples were dried at 105 °C to a constant weight to determine dry matter (DM) content for subsequent analysis. Crude protein (CP) was determined using a Kjeldahl apparatus (KDN-2008, Shanghai Xianjian, China). NDF and acid detergent fiber (ADF) contents were analyzed using a fiber analyzer (ANKOM-A2000i, Ankom Technology, United States). Ether extract (EE) was determined using a fat analyzer (SZC-C, Shanghai Xianjian, China). Gross energy (GE) and fecal energy (FE) were measured using an oxygen bomb calorimeter (Parr6400, Parr Instrument Company, Champaign, IL, United States). Nutrient apparent digestibility and digestive energy (DE) were calculated using the following formulas:


$$\begin{array}{l} \text{Apparent digestibility}\;(\%)\\ =((\text{Nutrient in feed}-\text{Nutrient in feces})/\text{Nutrient in feed})\times 100 \end{array}$$

$$\begin{array}{c} \text{Digestive energy}\;(\mathrm{MJ}/\mathrm{kg})=\text{Intake gross energy}\;(\mathrm{GE})\\ -\text{Fecal energy}\;(\mathrm{FE}) \end{array}$$

### 2.6 DNA extraction and high-throughput sequencing

The 18 samples collected were sent to Rhonin Bioscience Co., Ltd. (Chengdu, Sichuan, China) for metagenomic sequencing and functional analysis. Microbial genomic DNA was extracted from each sample using a PowerFecalTM Fecal DNA Kit (MOBIO, Carlsbad, CA, United States) following the manufacturer’s instructions. The sample gDNA was purified using Zymo Research BIOMICS DNA Microprep Kit (Cat# D4301, United States). The quality of the extracted DNA was assessed using fluorescence quantitative PCR and agarose gel electrophoresis. After quality control, the DNA was utilized to construct sequencing libraries with the TruSeq™ DNA PCR-Free Sample Prep Kit (Illumina, Inc., San Diego, CA, United States) following the manufacturer’s protocols. Second-generation sequencing (Next-Generation Sequencing, NGS) was performed using the PE150 sequencing method on the Illumina NovaSeq 6,000 (Illumina, Inc., San Diego, CA, United States). The obtained data underwent quality control and were used for bioinformatics analysis.

### 2.7 Taxonomy profiling

Raw data were preprocessed using Trimmomatic (version 0.36) (12) to obtain high-quality sequences. Kraken2 was used for taxonomic annotation of each sequence (13), and species counts were compiled to construct an operational taxonomic unit (OTU) table. Using R software (version 4.4.2), the abundance data were statistically analyzed at taxonomic levels, which culminated in species composition and abundance distribution results. *α* diversity indices, including Shannon, Simpson, Chao1, and ACE, were calculated using the “Vegan” package (version 2.6.2) in R software (version 4.4.2) to evaluate species diversity and richness within each sample’s gut microbiota (14). *β* diversity was assessed using Bray–Curtis distance algorithms with the “Vegan” package in R software (version 4.4.2) to obtain distance matrices for principal coordinates analysis (PCoA) (15). The adonis function was used to perform non-parametric multivariate analysis of variance (PERMANOVA) (16) in order to examine the significance of similarities and differences in the structure of gut microbial community among the samples and groups.

### 2.8 Assembly and gene prediction

The clean reads obtained after quality control were used for metagenomic assembly with SPAdes (17), and the resulting contigs were used to evaluate the assembly effect using QUAST (18). Gene prediction was performed on the assembled contigs using Prodigal (version 2.6.3) (19), and the predicted nucleic acid sequence and amino acid sequence of the coding gene were obtained. Non-redundant gene clusters were generated using MMseqs2 (version 13.45111) (20), with the following parameters: identity > = 95% and coverage > = 90 as the clustering threshold and the longest sequence selected as the representative sequence. All representative sequences formed a non-redundant gene set for subsequent quantitative analysis. The non-redundant gene sets obtained were annotated using the KEGG (21, 22) and CAZy (23) functional databases and were statistically analyzed in combination with the quantitative results of the genes. Linear discriminant analysis effect size (LEfSe) analysis of CAZy families was performed using the LEfSe online platform.^1^

### 2.9 Data analysis

All experiment results had at least three replicates and were expressed as mean ± standard error (SEM). ANOVA (SPSS 20.0) was used for statistical analysis, and a *p*-value of < 0.05 was considered statistically significant.

## 3 Result

### 3.1 Intake, digestibility, and digestive energy

As shown in Table 2, the DM digestibility of the L group was higher (*p* < 0.05) compared to the H group. Moreover, the FMD fed with medium NDF levels had significantly higher (*p* < 0.05) NDF and ADF apparent digestibility compared to the H group. Similarly, NDF digestibility initially increased but then declined with increasing NDF levels. Nevertheless, there were no significant differences in DMI, OM digestibility, and DE among the three experimental groups (*p* > 0.05).

**Table 2:** Effects of the different NDF levels on feed intake, apparent digestibility, and digestible energy.

Item	Group			*P*-value
	L	M	H	
DMI (g/d)	405.48 ± 87.58	482.69 ± 121.35	410.77 ± 116.95	0.191
DM (%)	73.76 ± 1.31^a^	71.19 ± 1.08^ab^	70.19 ± 0.57^b^	0.079
OM (%)	75.85 ± 1.46	73.97 ± 1.10	73.14 ± 0.47	0.237
CP (%)	59.50 ± 1.83	59.96 ± 1.37	59.94 ± 0.97	0.969
NDF (%)	44.63 ± 1.36^ab^	50.18 ± 2.46^a^	41.42 ± 1.56^b^	0.018
ADF (%)	45.01 ± 2.03^ab^	50.11 ± 2.60^a^	40.44 ± 1.44^b^	0.021
DE (MJ/kg)	11.76 ± 0.25	11.45 ± 0.19	11.37 ± 0.10	0.354

L = low NDF (21.6%); M = middle NDF (25.14%); H = high NDF (28.47%); DMI = dry matter intake; DM = dry matter; OM = organic matter; CP = crude protein; NDF = neutral detergent fiber; ADF = acid detergent fiber; DE = digestible energy. Within a row, values with different lowercase letters indicate significant differences among groups (*p* < 0.05).

### 3.2 Profiling of the intestinal metagenome

Metagenome sequencing analysis was performed on the fecal samples obtained from the 18 FMD, and a total of 630,074,687 raw reads were obtained. After filtering the host genome data, 628,138,962 clean reads were obtained, averaging 34,896,609 per sample.

**Figure 1:**
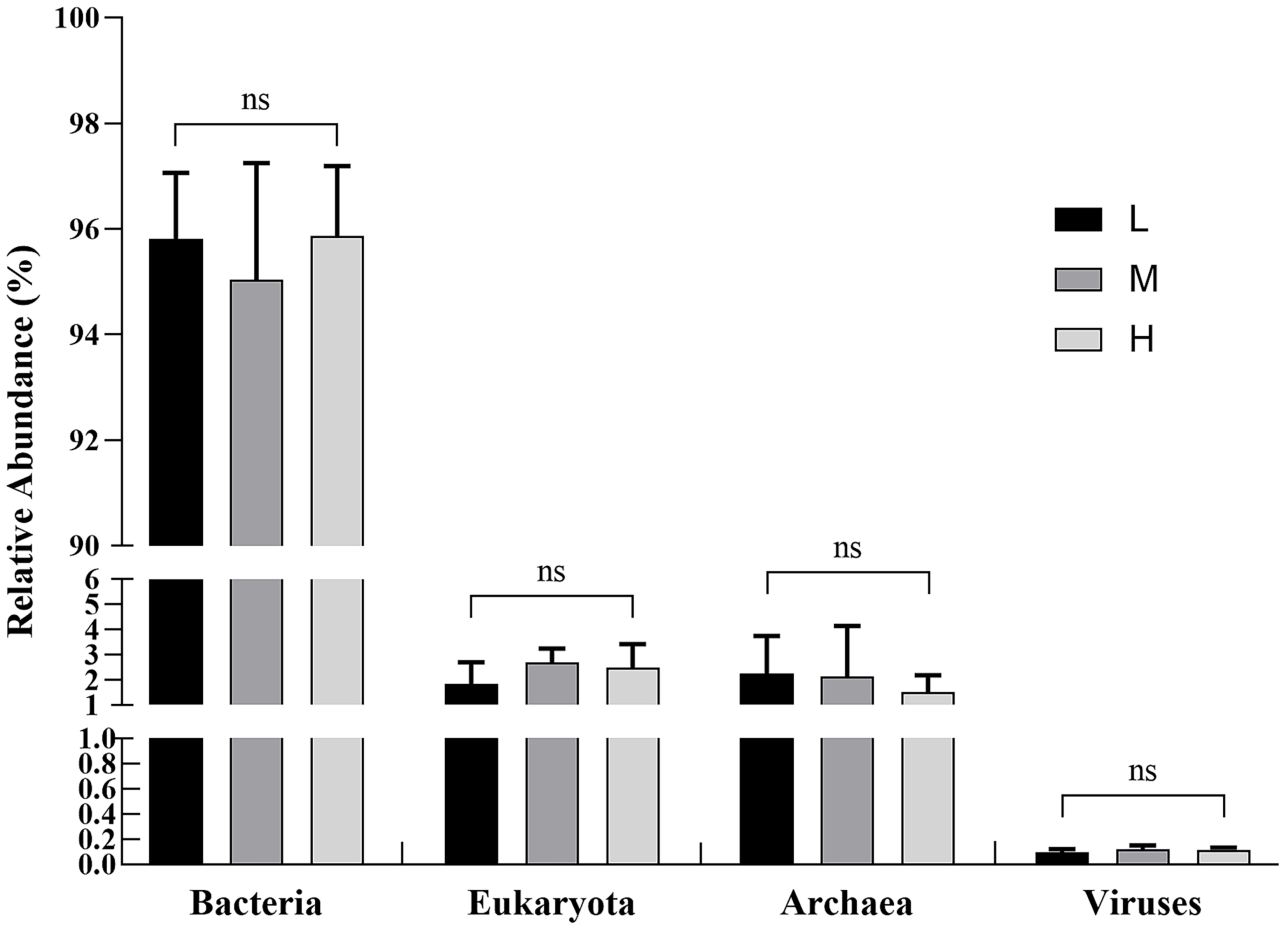
Influence of the different NDF levels on relative abundance at the intestinal kingdom level. L = low NDF (21.6%); M = middle NDF (25.14%); H = high NDF (28.47%). The “ns” indicates that the difference was not significant (*p* > 0.05).

A total of 4 kingdoms, 82 phyla, 178 classes, 338 orders, 764 families, and 3,081 genera were obtained. At the kingdom level, the relative abundance, from highest to lowest, was as follows: Bacteria (90.69 ~ 97.74%), eukaryotes (1.24 ~ 3.79%), archaea (0.57 ~ 4.29%), and viruses (0.07 ~ 0.18%). In addition, no significant differences were observed among the three treatments (*p* > 0.05) (Figure 1).

### 3.3 Comparison of intestinal microbial diversity

**Figure 2:**
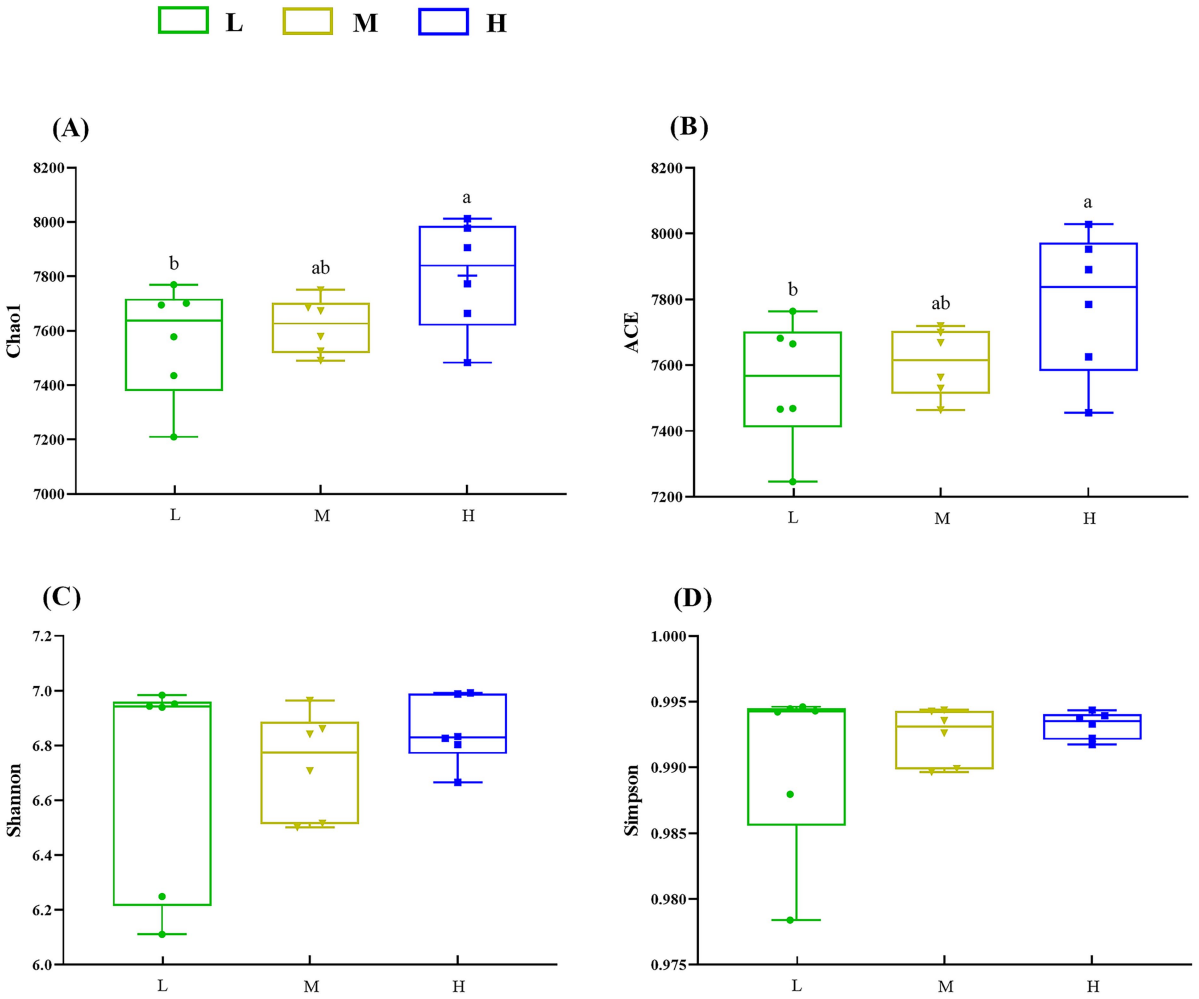
Influence of different NDF levels on α diversity. **(A)** Chao1, **(B)** ACE, **(C)** Shannon, and **(D)** Simpson. Different lowercase letters denote significant differences among groups (*p* < 0.05). L = low NDF (21.6%); M = middle NDF (25.14%); H = high NDF (28.47%).

**Figure 3:**
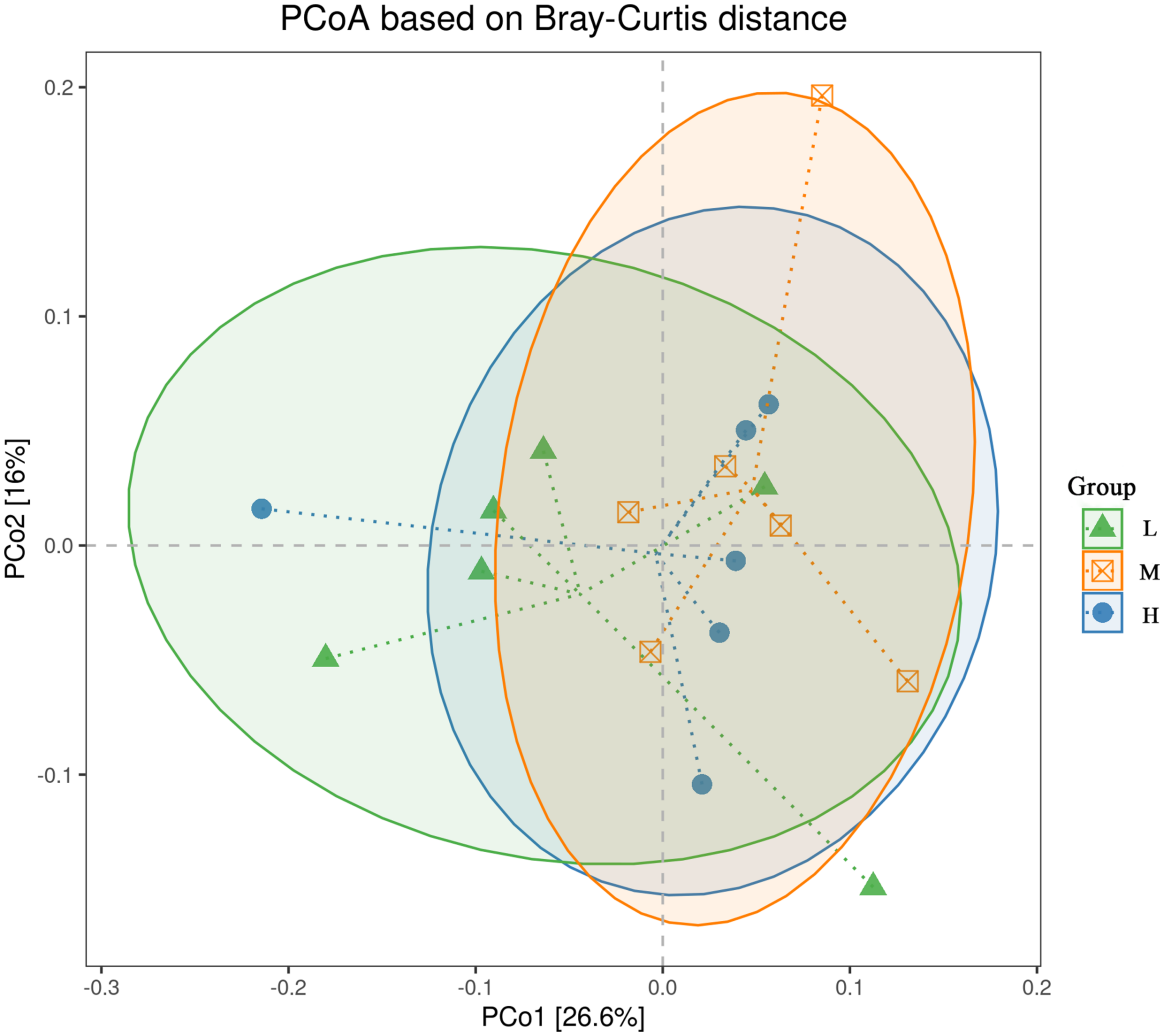
Influence of the different NDF levels on β diversity. L = low NDF (21.6%); M = middle NDF (25.14%); H = high NDF (28.47%).

In the *α*-diversity analysis results (Figure 2), the Chao1 (Figure 2A) and ACE (Figure 2B) indices in the H group were higher (*p* < 0.05) than those in the L group. However, no significant difference (*p* > 0.05) for the Shannon (Figure 2C) and Simpson (Figure 2D) indices was found among the three groups. Furthermore, PERMANOVA of *β* diversity revealed no significant differences in fecal microbial communities among the three groups (*p* > 0.05), although a trend toward a difference was observed between the L and M groups (*p* = 0.067) (Figure 3; Table 3).

**Table 3:** PERMANOVA of the gut microbiota under the different NDF levels.

Group	R^2^	*P*-value
L vs. M	0.129	0.067
L vs. H	0.080	0.554
M vs. H	0.069	0.814
L vs. M vs. H	0.122	0.380

L = low NDF (21.6%); M = middle NDF (25.14%); H = high NDF (28.47%).

### 3.4 Comparison of intestinal microbiome

**Figure 4:**
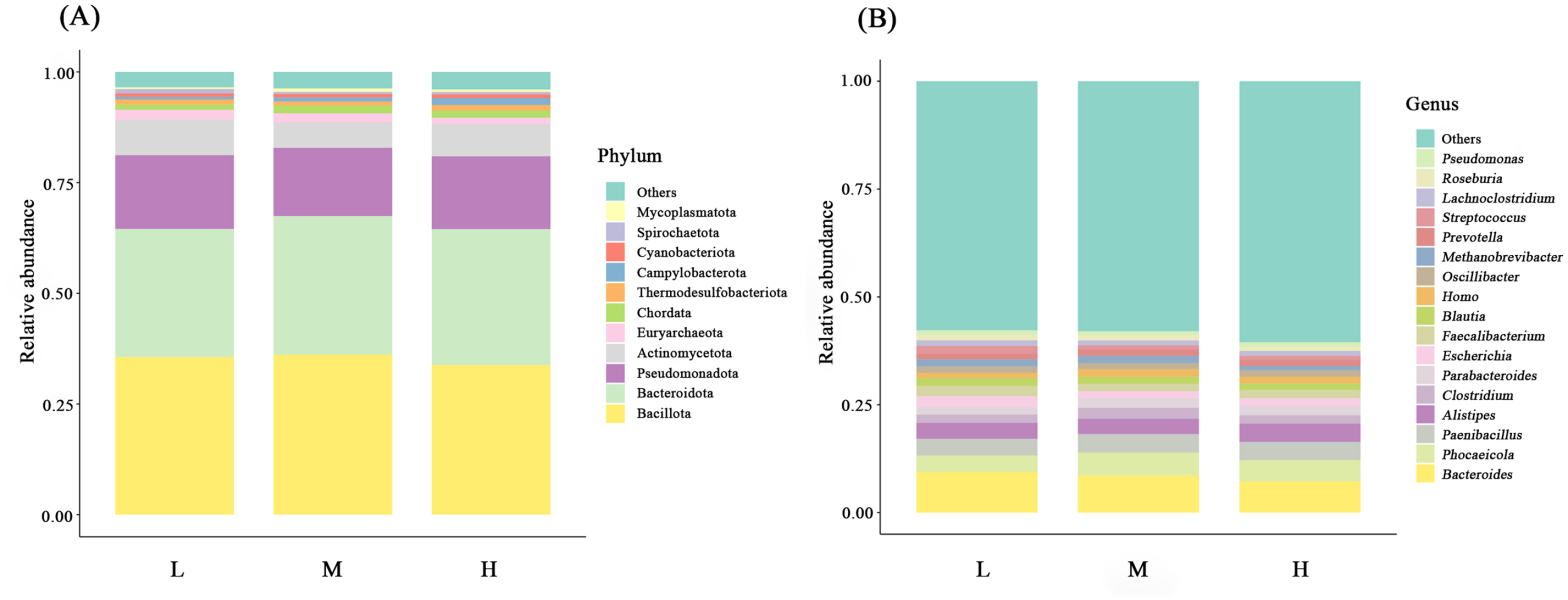
Bar charts showing the relative abundance of the intestinal microbiota at the phylum classification level **(A)** and the genus classification level **(B)**. L = low NDF (21.6%); M = middle NDF (25.14%); H = high NDF (28.47%).

At the phylum level, the dominant phyla across the three groups included Bacillota (L = 35.62%; M = 36.16%; H = 33.83%) and Bacteroidota (L = 28.93%; M = 31.30%; H = 30.69%), followed by Pseudomonadota (L = 16.65%; M = 15.42%; H = 16.44%), Actinomycetota (L = 8.08%; M = 5.74%; H = 7.27%), Euryarchaeota (L = 2.17%; M = 2.04%; H = 1.43%), and Chordata (L = 1.20%; M = 1.77%; H = 1.63%) (Figure 4A; Supplementary Table S1). With an increase in the NDF level, the relative abundance of Bacillota and Bacteroidota first increased and then decreased. There was no significant difference in microbial relative abundance among the three groups (*p* > 0.05). In addition, the relative abundance of Mycoplasmatota in the M group was significantly higher compared to the L group (*p* < 0.05).

At the genus level, species with over 1% average relative abundance in gut microbial communities among the three groups are presented (Figure 4B; Supplementary Table S2). Among these genera, *Bacteroides* (L = 9.34%; M = 7.97%; H = 8.78%) exhibited the highest relative abundance, followed by *Phocaeicola* (L = 3.89%; M = 4.08%; H = 4.69%) and *Paenibacillus* (L = 3.90%; M = 4.33%; H = 4.59%). However, no significant differences in the relative abundance at the genus level were observed among the three treatments (*p* > 0.05).

### 3.5 Functional gene annotation analysis

#### 3.5.1 KEGG functional annotation

**Figure 5:**
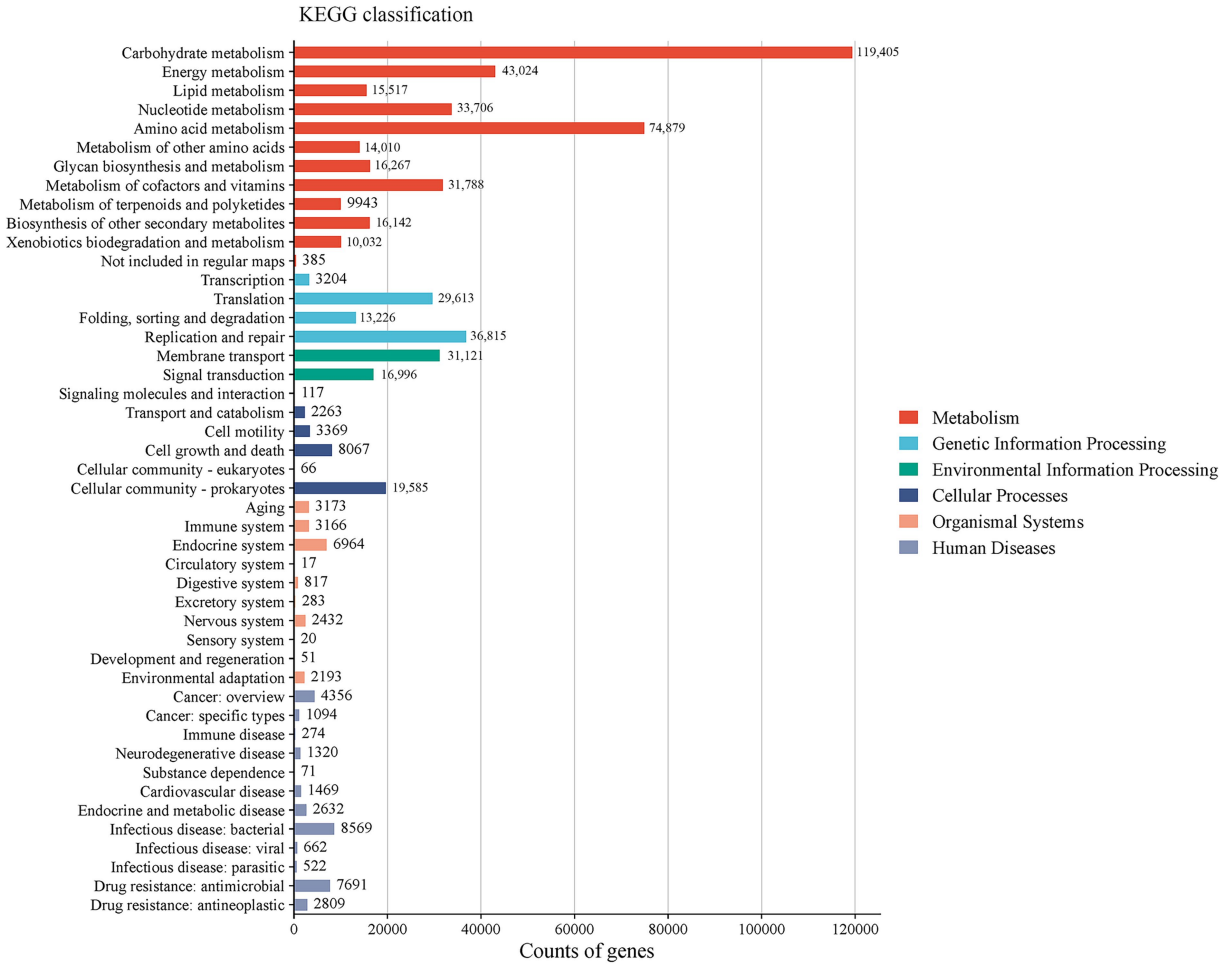
Column chart of annotation classification based on the KEGG database (Level 1 and Level 2).

The KEGG database was used to annotate the pathways of different levels and abundances. A total of 1,048,575 UniGenes were annotated, covering 426 pathways across six major categories. The categories ranked by the relative abundance of enriched genes from highest to lowest were as follows (Figure 5): metabolism (64.19%), genetic information processing (13.81%), environmental information processing (8.02%), cellular processes (5.55%), organismal systems (3.19%), and human diseases (5.24%). The level 2 classification results showed that carbohydrate metabolism (19.89%) had the highest relative abundance, followed by amino acid metabolism (12.48%), energy metabolism (7.17%), replication and repair (6.12%), and metabolism of cofactors and vitamins (5.31%).

**Figure 6:**
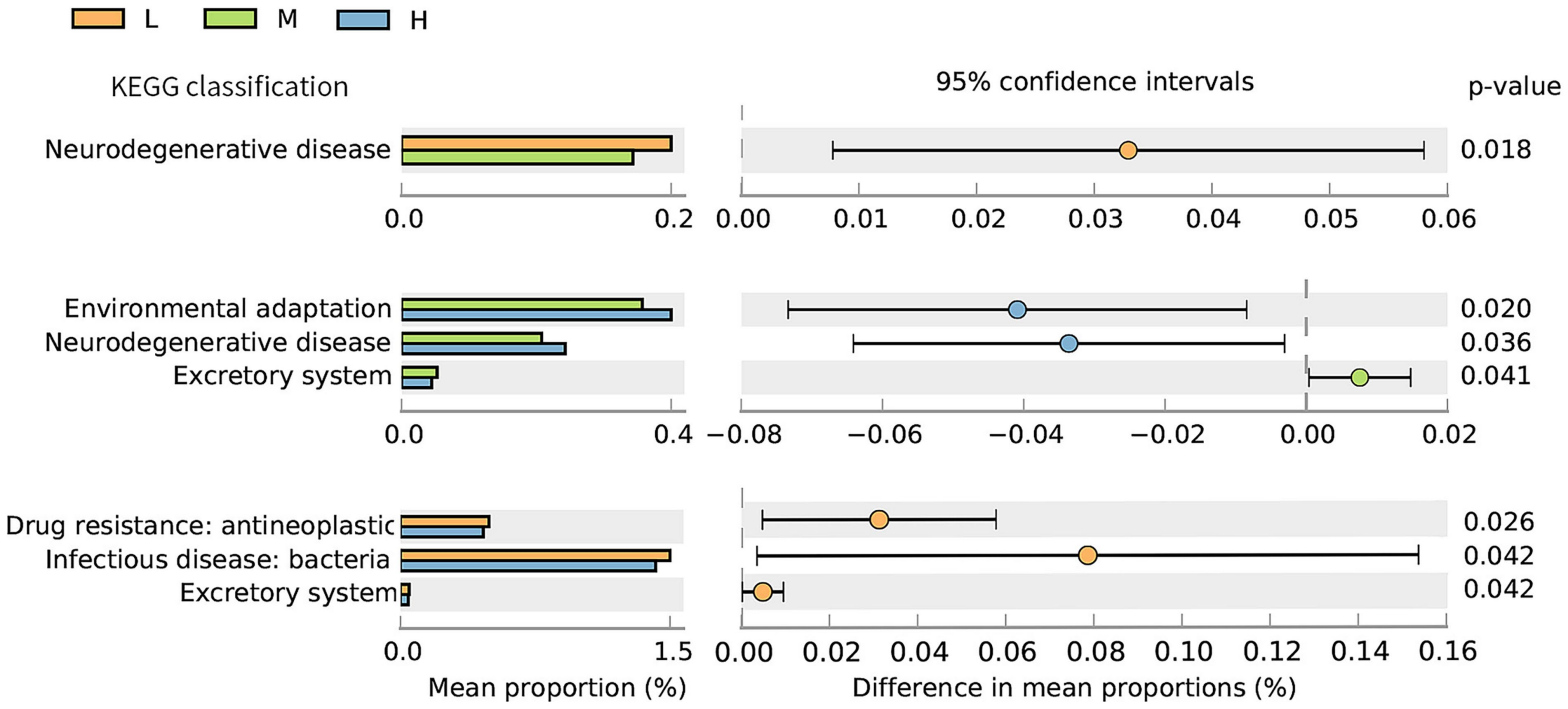
Differential analysis diagram of the KEGG functional annotations (Level 2) under the different NDF levels. L = low NDF (21.6%); M = middle NDF (25.14%); H = high NDF (28.47%).

The distribution plot of the relative abundance differences in the KEGG functional annotation is shown in Figure 6. The relative abundance of neurodegenerative disease in the L group was significantly higher (*p* < 0.05) than in the M group. In addition, the abundances of drug resistance: antineoplastic, infectious disease: bacterial, and excretory system in the L group were significantly higher (*p* < 0.05) than those in the H group. The abundances of environmental adaptation and neurodegenerative disease were significantly lower (*p* < 0.05) in the M group compared to the H group, while the abundance of the excretory system was higher than that in the H group (*p* < 0.05).

#### 3.5.2 CAZy functional annotation

**Figure 7:**
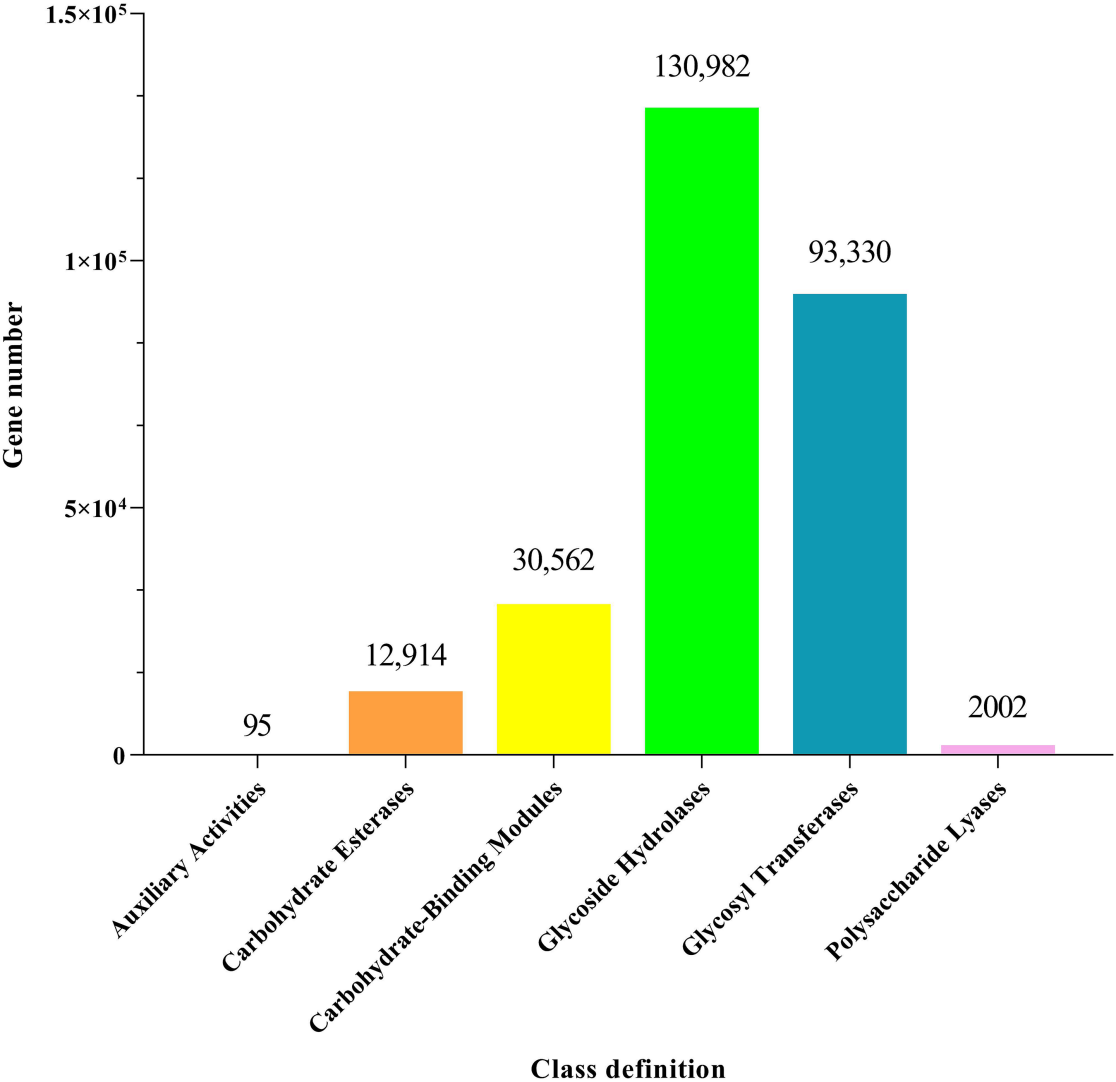
Bar chart showing the number of carbohydrate-active enzyme genes annotated based on the CAZy database.

The column chart illustrating the number of carbohydrate-active enzyme genes was generated by comparison with the CAZy database. As shown in Figure 7, a total of 269,885 carbohydrate-active enzymes were predicted. Among the six types of enzyme molecules, glycoside hydrolases (GHs) accounted for the highest proportion at 48.53%, while auxiliary oxidoreductases (AAs) accounted for the lowest proportion at 0.04%.

**Figure 8:**
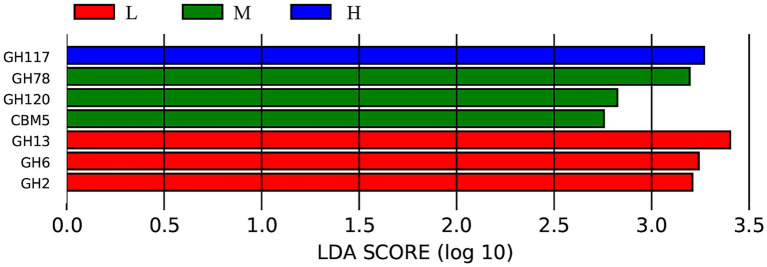
LEfSe analysis bar chart showing the differences in the carbohydrate-active enzymes of the intestinal microbiota. L = low NDF (21.6%); M = middle NDF (25.14%); H = high NDF (28.47%).

To further analyze the differences in carbohydrate-active enzymes caused by varying NDF levels, the LEfSe analysis results shown in Figure 8 indicated that the LDA scores of seven biomarkers among the three groups were greater than 2. Notably, according to the LDA scores, GH177 was significantly enriched in the H group (LDA ≥ 2; *p* < 0.05), while GH78, GH120, and GBM5 were enriched in the M group (LDA ≥ 2; *p* < 0.05). In contrast, GH13, GH6, and GH2 showed significant enrichment in the L group (LDA ≥ 2; *p* < 0.05).

## 4 Discussion

To ensure experimental precision, this study employed a TMR feeding system instead of the conventional separate feeding of concentrate and roughage. The palatability of the TMR influenced intake patterns, although no statistically significant differences in DMI were observed across the NDF levels, and it is possible that when the NDF content exceeds a certain level, it may enhance the animal’s satiety, thereby reducing DMI. Notably, the M group exhibited the highest DMI values, suggesting a potential acceptability for balancing fiber content and feed intake. Nutrient digestibility reflects the efficiency of nutrient utilization by animals, and high nutrient digestibility is beneficial for growth performance. Although the L group exhibited higher DM digestibility, its DMI was low. The elevated DM digestibility compared to the H group may be attributed to the lower NDF content, which allowed rumen microbes—normally adherent to fiber—to preferentially ferment carbohydrates and proteins, thereby contributing to the higher DM digestibility in the L group. Similarly, a study in cattle reported that the low-fiber group exhibited higher DM digestibility but lower NDF digestibility compared to the high-fiber group (24). In addition, the digestibility of NDF showed significant quadratic relationships with increasing dietary NDF levels. The quadratic regression analysis of the NDF levels and NDF digestibility showed that the regression equation was Y = − 0.761 × X^2^ + 37.188 × X − 400.781 (R^2^ = 0.549). In the formula, Y is NDF digestibility and X is the NDF level, and maximum predicted NDF digestibility occurred at an NDF level of 24.43%. With reference to previous studies in other ruminant species, the dietary NDF level for Tarim red deer was 37 ~ 47% (25). For growing lambs, an NDF level of 280 g/kg of ration was suggested (26). In peak-lactation dairy cows, a dietary NDF level of 28% was recommended (27). In contrast, as a specialized small ruminant with distinct browsing preferences (*Moschus berezovskii* preferentially consumes tender leaves while avoiding graminoids), the FMD exhibits markedly lower fiber tolerance. This dietary behavior helps explain why the experimentally determined NDF level, based on NDF digestibility, was lower compared to other herbivores.

The gastrointestinal microbiome constitutes a fundamental component of ruminant physiology, playing a key role in nutrient digestion and utilization, host immunity, intestinal barrier maintenance, and intestinal epithelium differentiation (10, 28). In this study, the *α*-diversity analysis revealed significantly higher Chao 1 and ACE indices in the H group compared to the L group, and the PERMANOVA revealed a tendency for separation between the L group and M group, indicating that gut microbial richness progressively increased with elevated dietary NDF levels. Zhang et al. (29) observed that rumen microbial diversity had a positive association with NDF levels. The H group’s diet, characterized by higher structural carbohydrate content, presumably enhanced rumen distension, and this dietary composition likely led to increased retention of undigested structural carbohydrates in the rumen. The associated ruminal microbiota, adhering to these fibrous substrates, were subsequently conveyed to the intestinal tract via the digesta passage, which may explain the demonstrated elevation in microbial α diversity. The microbial community was predominantly composed of Bacillota and Bacteroidetes, which were the dominant phyla, consistent with previous findings in FMD (30, 31). Bacillota, as primary cellulolytic bacteria, degrade fiber into short-chain fatty acids (SCFAs) for host utilization, while Bacteroidetes specialize in carbohydrate and protein degradation, promoting gastrointestinal immune system development (32, 33). Although the differences were not statistically significant, the M group exhibited the highest relative abundance of these two predominant bacterial phyla. Evidently, Mycoplasmatota (formerly Tenericutes) showed significantly higher relative abundance in the M group than in the L group. Similar to our findings, a significant quadratic correlation was observed between physically effective NDF (peNDF_1.18_) levels and Tenericutes abundance in the rumen of goats (34). Moreover, dietary fiber supplementation was associated with increased Tenericutes abundance in the porcine cecum (35). As cell-wall-deficient bacteria, Mycoplasmatota may exhibit unique interactions with the host intestinal epithelium (36). Marine studies further suggest its potential involvement in DNA metabolism (37, 38). However, its ecological role in mammalian gut systems remains poorly characterized. Therefore, further investigation is required to elucidate whether Mycoplasmatota participates in fiber degradation. In mammalian systems, although transient dietary alterations provoke immediate microbial shifts, attainment of a stabilized gut microbiota configuration generally requires an extended adaptation period that ranges from 30 days to multiple months (39, 40). The absence of intergroup differences at the genus level may reflect the limitation of the 50-day experimental period; however, subsequent metagenomic functional analyses revealed distinct characteristics among the three groups.

KEGG pathway analysis demonstrated that metabolism dominated the functional potential of the gut microbiota, whereas organismal systems were minimally represented, consistent with previous findings (41). Among the three treatment groups, differences in the KEGG pathways were primarily observed in organismal systems and human disease categories. The significant enrichment of the human disease pathways in the L group suggested a potential predisposition to disease development or manifestation of inflammatory conditions. Interestingly, the H group exhibited environmental adaptation enrichment under high NDF levels and low DMI conditions. This phenomenon may be attributed to the animal’s adaptive self-regulation in response to stress induced by external factors under conditions of reduced total energy intake. A previous study indicated that intestinal barrier disruption and gut microbiota dysbiosis are associated with reduced activity of excretory system pathways in mouse models (42). Consistently, the enrichment of the excretory system implied that the moderate dietary NDF level could enhance digestive and metabolic efficiency in FMD, potentially optimizing digestion and waste excretion processes. LEfSe analysis based on the CAZy database identified differential microbial biomarkers across the three treatment groups, with the majority belonging to GHs. All known agar-degrading bacteria possess at least one conserved GH117 enzyme, which is essential for their polysaccharide utilization capabilities (43). GH78 has been demonstrated to hydrolyze ester bonds between lignin and polysaccharides in plant cell walls, and it exhibits robust *α*-L-rhamnosidase activity, which is implicated in the degradation of hemicellulose and pectin chains (44). GH120 functions as a *β*-xylosidase, catalyzing the hydrolysis of xylooligosaccharides into monomeric xylose units (45, 46). Carbohydrate-binding modules (CBMs) primarily facilitate substrate conversion, with CBM5 specifically acting as a chitinase-binding module that enhances chitinase activity on fermentative substrates (47). The GH13 family includes starch-debranching enzymes that specifically and efficiently hydrolyze α-1,6-glycosidic bonds at starch branching points, thereby enhancing starch utilization efficiency (48). GH6 comprises glucanases and cellobiohydrolases, constituting a specialized enzyme family involved in cellulose degradation (49). GH2 plays a pivotal role in oligosaccharide degradation (50). These results indicate that the L group was primarily involved in degrading non-structural carbohydrates (e.g., starch and oligosaccharides), whereas the M group predominantly targeted the fibrous components of plant cell walls, which aligns with the high NDF digestibility observed in this study.

## 5 Conclusion

Based on the apparent nutrient digestibility results, an NDF level of 24.43% is recommended for optimal fiber degradation in FMD. Gut microbiome structure and function analysis further demonstrated that the M group (NDF level of 25.14%) exhibited superior digestive system performance, with higher enzymatic activity for structural carbohydrate degradation. Therefore, we recommend maintaining dietary NDF at approximately 25% for adult male FMD under the conditions of this experiment.

## Data Availability

Raw sequencing reads have been deposited into the National Center for Biotechnology Information Sequence Read Archive (SRA) database (accession number: PRJNA1266873).

## Footnotes

^1^ https://huttenhower.sph.harvard.edu/galaxy/
