# Supplementary material for: Effects of neutral detergent fiber levels on apparent nutrient digestibility and intestinal microbiota composition and function in forest musk deer
Source: Front Vet Sci. 2025 Sep 11;12:1658189. doi: 10.3389/fvets.2025.1658189 (PMC12460106; doi:10.3389/fvets.2025.1658189)
Supplement: Supplementary file 1 [file Table_1.DOCX]

Supplementary Material

# Supplementary TABLE S1 Relative abundance of gut microorganisms at phylum level (%).

| Phylum | Group | | | p value |
| --- | --- | --- | --- | --- |
|  | L | M | H |  |
| Bacillota | 35.62 ± 1.74 | 36.16 ± 3.00 | 33.83 ± 1.56 | 0.740 |
| Bacteroidota | 28.93 ± 2.86 | 31.30 ± 2.55 | 30.69 ± 3.07 | 0.830 |
| Pseudomonadota | 16.65 ± 1.91 | 15.42 ± 2.46 | 16.44 ± 1.40 | 0.895 |
| Actinomycetota | 8.08 ± 1.46 | 5.74 ± 0.41 | 7.27 ± 0.84 | 0.275 |
| Euryarchaeota | 2.17 ± 0.61 | 2.04 ± 0.82 | 1.43 ± 0.26 | 0.661 |
| Chordata | 1.20 ± 0.23 | 1.77 ± 0.13 | 1.63 ± 0.25 | 0.170 |
| Thermodesulfobacteriota | 1.17 ± 0.12 | 0.91 ± 0.06 | 1.27 ± 0.25 | 0.304 |
| Campylobacterota | 0.59 ± 0.08 | 0.89 ± 0.12 | 1.52 ± 0.70 | 0.303 |
| Cyanobacteriota | 0.75 ± 0.05 | 0.80 ± 0.03 | 0.87 ± 0.05 | 0.162 |
| Spirochaetota | 1.00 ± 0.38 | 0.48 ± 0.04 | 0.55 ± 0.05 | 0.232 |
| Mycoplasmatota | 0.38 ± 0.08^b^ | 0.77 ± 0.15^a^ | 0.54 ± 0.10^ab^ | 0.090 |

L = low NDF (21.6%); M = middle NDF (25.14%); H = high NDF (28.47%).

# Supplementary TABLE S2 Relative abundance of gut microorganisms at genus level (%).

| Genus | Group | | | p value |
| --- | --- | --- | --- | --- |
|  | L | M | H |  |
| Bacteroides | 9.34 ± 1.52 | 7.97 ± 0.80 | 8.78 ± 0.98 | 0.394 |
| Phocaeicola | 3.89 ± 0.25 | 4.08 ± 0.30 | 4.69 ± 0.65 | 0.201 |
| Paenibacillus | 3.90 ± 0.40 | 4.33 ± 0.34 | 4.59 ± 0.12 | 0.688 |
| Alistipes | 3.70 ± 0.97 | 2.84 ± 0.41 | 3.10 ± 0.39 | 0.863 |
| Clostridium | 1.88 ± 0.25 | 2.14 ± 0.36 | 2.17 ± 0.35 | 0.238 |
| Parabacteroides | 1.73 ± 0.21 | 1.63 ± 0.17 | 1.99 ± 0.39 | 0.411 |
| Escherichia | 2.60 ± 2.07 | 2.64 ± 2.06 | 0.55 ± 0.12 | 0.860 |
| Faecalibacterium | 2.31 ± 0.29 | 2.45 ± 0.21 | 2.20 ± 0.28 | 0.134 |
| Blautia | 1.84 ± 0.16 | 1.88 ± 0.15 | 1.66 ± 0.19 | 0.432 |
| Homo | 1.20 ± 0.23 | 1.36 ± 0.25 | 1.56 ± 0.24 | 0.170 |
| Oscillibacter | 1.51 ± 0.16 | 1.65 ± 0.08 | 1.54 ± 0.11 | 0.904 |
| Methanobrevibacter | 1.61 ± 0.67 | 1.01 ± 0.52 | 1.31 ± 0.82 | 0.693 |
| Prevotella | 1.33 ± 0.15 | 1.32 ± 0.15 | 1.54 ± 0.16 | 0.664 |
| Streptococcus | 1.83 ± 1.05 | 0.79 ± 0.06 | 0.78 ± 0.06 | 0.549 |
| Lachnoclostridium | 1.29 ± 0.12 | 1.36 ± 0.10 | 1.20 ± 0.13 | 0.534 |
| Roseburia | 1.25 ± 0.21 | 1.25 ± 0.21 | 1.11 ± 0.24 | 0.549 |
| Pseudomonas | 1.07 ± 0.13 | 1.12 ± 0.10 | 1.12 ± 0.10 | 0.277 |

# L = low NDF (21.6%); M = middle NDF (25.14%); H = high NDF (28.47%).
